# Benzoic acid–2,9-dimethyl­phenanthroline (1/1)

**DOI:** 10.1107/S1600536810029065

**Published:** 2010-07-24

**Authors:** Hadi D. Arman, Trupta Kaulgud, Edward R. T. Tiekink

**Affiliations:** a Department of Chemistry, The University of Texas at San Antonio, One UTSA Circle, San Antonio, Texas 78249-0698, USA; b Department of Chemistry, University of Malaya, 50603 Kuala Lumpur, Malaysia

## Abstract

The constituents of the title 1:1 co-crystal, C_7_H_6_O_2_·C_14_H_12_N_2_, are connected into dimeric aggregates by a bifurcated O—H⋯N hydrogen bond; the hydroxyl-H atom is hydrogen bonded to the two N atoms of the 2,9-dimethyl­phenanthroline. The hydrogen-bonded residues are almost orthogonal to each other [dihedral angle = 78.56 (7) °]. In the crystal packing, the aggregates are assembled into layers in the *bc* plane by π⋯π inter­actions [ring centroid⋯ring centroid distance = 3.5577 (16) Å] involving the pyridyl rings, and C–H⋯π contacts involving the phenanthroline-H atom and the phenyl ring of the acid.

## Related literature

For related studies on co-crystal formation, see: Broker & Tiekink (2007 ▶); Broker *et al.* (2008 ▶).
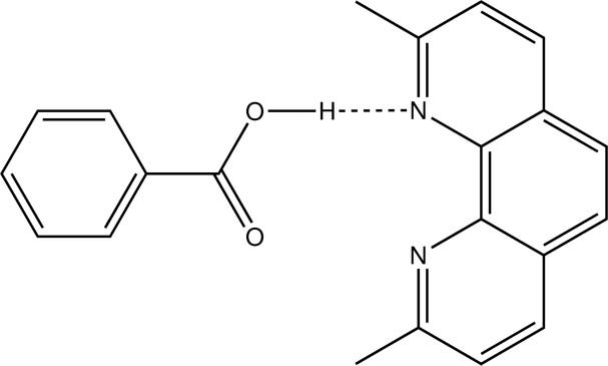


## Experimental

### 

#### Crystal data

C_7_H_6_O_2_·C_14_H_12_N_2_
*M* *_r_* = 330.37
Monoclinic, 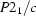

*a* = 13.575 (5) Å
*b* = 11.645 (4) Å
*c* = 11.148 (4) Å
β = 104.832 (6)°
*V* = 1703.6 (11) Å^3^
*Z* = 4
Mo *K*α radiation
μ = 0.08 mm^−1^
*T* = 98 K
0.46 × 0.31 × 0.20 mm

#### Data collection

Rigaku AFC12/SATURN724 diffractometer
Absorption correction: multi-scan (*ABSCOR*; Higashi, 1995 ▶) *T*_min_ = 0.864, *T*_max_ = 1
13167 measured reflections
3907 independent reflections
3589 reflections with *I* > 2σ(*I*)
*R*_int_ = 0.035

#### Refinement

*R*[*F* ^2^ > 2σ(*F* ^2^)] = 0.060
*wR*(*F* ^2^) = 0.156
*S* = 1.09
3907 reflections
231 parameters
1 restraint
H atoms treated by a mixture of independent and constrained refinement
Δρ_max_ = 0.38 e Å^−3^
Δρ_min_ = −0.55 e Å^−3^

### 

Data collection: *CrystalClear* (Molecular Structure Corporation & Rigaku, 2005 ▶); cell refinement: *CrystalClear*; data reduction: *CrystalClear*; program(s) used to solve structure: *SHELXS97* (Sheldrick, 2008 ▶); program(s) used to refine structure: *SHELXL97* (Sheldrick, 2008 ▶); molecular graphics: *ORTEP-3* (Farrugia, 1997 ▶) and *DIAMOND* (Brandenburg, 2006 ▶); software used to prepare material for publication: *publCIF* (Westrip, 2010 ▶).

## Supplementary Material

Crystal structure: contains datablocks I. DOI: 10.1107/S1600536810029065/bt5303sup1.cif

Structure factors: contains datablocks I. DOI: 10.1107/S1600536810029065/bt5303Isup2.hkl

Additional supplementary materials:  crystallographic information; 3D view; checkCIF report

## Figures and Tables

**Table 1:** Hydrogen-bond geometry (Å, °) *Cg* is the centroid of the C2–C7 ring.

*D*—H⋯*A*	*D*—H	H⋯*A*	*D*⋯*A*	*D*—H⋯*A*
O1—H1o⋯N1	0.84 (1)	2.33 (2)	2.973 (2)	134 (2)
O1—H1o⋯N2	0.84 (1)	2.09 (2)	2.788 (2)	141 (2)
C19—H19⋯*Cg*^i^	0.95	2.60	3.426 (2)	145

Symmetry code: (i) 
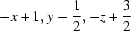
.

**Fig. 1.**
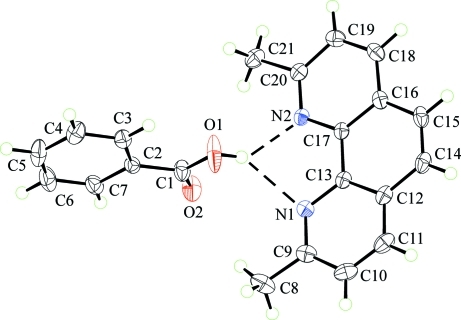
Molecular structure of (I) showing atom-labelling scheme and displacement ellipsoids at the 50% probability level. The O—H···N hydrogen bonds are shown as dashed lines.

**Fig. 2.**
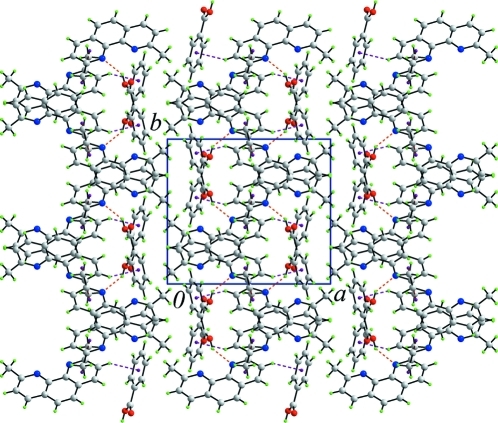
Stacking of layers along the a axis in (I). The O—H···N (orange), π···π (purple) and C–H···π (brown) contacts are shown as dashed lines.

## supplementary crystallographic information

### Comment

As a continuation of studies into the phenomenon of co-crystallization (Broker & Tiekink, 2007; Broker *et al.*, 2008), the co-crystallization of 2,9-dimethylphenanthroline and benzoic acid was investigated, leading to the isolation of the 1:1 co-crystal, (I).

The components of (I), Fig. 1, are connected by two O–H···N hydrogen bonds with the primary contact formed with the N2 atom with a weaker interaction to the the N1 atom, Table 1. The bifurcated nature of the O—H atom is responsible for the deviation of the O—H···N angles from 180 °. The carboxylic acid group is effectively co-planar with the benzene ring to which it is attached as seen in the O1—C1—C2—C3 torsion angle of 7.6 (2) °. The dihedral angle formed between the least-squares planes through the benzene ring and the 14 non-hydrogen atoms of the phenanthroline ring (r.m.s. deviation = 0.020 Å) is 78.56 (7) °, indicating an almost orthogonal relationship. The methyl-C8 and C21 atoms lie and 0.081 (2) and -0.032 (2) Å, respectively, out of the plane through the phenanthroline ring. In addition to the hydrogen bonding, π···π and C—H···π interactions are found in the crystal structure of (I). The former occur between centrosymmetrically related N2-pyridyl rings [*Cg*(N2,C16–C20)···*Cg*(N2,C16—C20)^i^ = 3.5577 (16) Å for *i*: 1 - *x*, 1 - *y*, 2 - *z*]. The C–H···π contact occurs between a phenanthroline-H and the benzene ring of the acid, Table 1. The result is the formation of layers that stack along the *a* axis, Fig. 2.

### Experimental

Colourless crystals of (I) were isolated from the 1/1 co-crystallization of 2,9-dimethylphenanthroline (ACROS; 0.08 mmol) and benzoic acid (Sigma-Aldrich; 0.07 mmol) in chloroform solution, m. pt. 399–403 K.

### Refinement

C-bound H-atoms were placed in calculated positions (C–H 0.95–0.98 Å) and were included in the refinement in the riding model approximation with *U*_iso_(H) set to 1.2–1.5*U*_eq_(C). The O-bound H-atom was located in a difference Fourier map and was refined with a distance restraint of O–H 0.840±0.001 Å, and with *U*_iso_(H) = 1.5*U*_eq_(O).

### Crystal data

**Table:**

C_7_H_6_O_2_·C_14_H_12_N_2_	*F*(000) = 696
*M**_r_* = 330.37	*D*_x_ = 1.288 Mg m^−^^3^
Monoclinic, *P*2_1_/*c*	Mo *K*α radiation, λ = 0.71073 Å
Hall symbol: -P 2ybc	Cell parameters from 6463 reflections
*a* = 13.575 (5) Å	θ = 2.1–40.6°
*b* = 11.645 (4) Å	µ = 0.08 mm^−^^1^
*c* = 11.148 (4) Å	*T* = 98 K
β = 104.832 (6)°	Block, colourless
*V* = 1703.6 (11) Å^3^	0.46 × 0.31 × 0.20 mm
*Z* = 4	

### Data collection

**Table:**

Rigaku AFC12K/SATURN724 diffractometer	3907 independent reflections
Radiation source: fine-focus sealed tube	3589 reflections with *I* > 2σ(*I*)
graphite	*R*_int_ = 0.035
ω scans	θ_max_ = 27.5°, θ_min_ = 2.3°
Absorption correction: multi-scan (*ABSCOR*; Higashi, 1995)	*h* = −17→17
*T*_min_ = 0.864, *T*_max_ = 1	*k* = −15→15
13167 measured reflections	*l* = −12→14

### Refinement

**Table:**

Refinement on *F*^2^	Primary atom site location: structure-invariant direct methods
Least-squares matrix: full	Secondary atom site location: difference Fourier map
*R*[*F*^2^ > 2σ(*F*^2^)] = 0.060	Hydrogen site location: inferred from neighbouring sites
*wR*(*F*^2^) = 0.156	H atoms treated by a mixture of independent and constrained refinement
*S* = 1.09	*w* = 1/[σ^2^(*F*_o_^2^) + (0.0717*P*)^2^ + 0.8352*P*] where *P* = (*F*_o_^2^ + 2*F*_c_^2^)/3
3907 reflections	(Δ/σ)_max_ = 0.001
231 parameters	Δρ_max_ = 0.38 e Å^−^^3^
1 restraint	Δρ_min_ = −0.55 e Å^−^^3^

### Special details

**Table:**

Geometry. All e.s.d.'s (except the e.s.d. in the dihedral angle between two l.s. planes) are estimated using the full covariance matrix. The cell e.s.d.'s are taken into account individually in the estimation of e.s.d.'s in distances, angles and torsion angles; correlations between e.s.d.'s in cell parameters are only used when they are defined by crystal symmetry. An approximate (isotropic) treatment of cell e.s.d.'s is used for estimating e.s.d.'s involving l.s. planes.
Refinement. Refinement of *F*^2^ against ALL reflections. The weighted *R*-factor *wR* and goodness of fit *S* are based on *F*^2^, conventional *R*-factors *R* are based on *F*, with *F* set to zero for negative *F*^2^. The threshold expression of *F*^2^ > σ(*F*^2^) is used only for calculating *R*-factors(gt) *etc*. and is not relevant to the choice of reflections for refinement. *R*-factors based on *F*^2^ are statistically about twice as large as those based on *F*, and *R*- factors based on ALL data will be even larger.

### Fractional atomic coordinates and isotropic or equivalent isotropic displacement parameters (Å^2^)

**Table:**

	*x*	*y*	*z*	*U*_iso_*/*U*_eq_	
O1	0.25983 (16)	0.62242 (12)	0.73806 (12)	0.0586 (5)	
H1O	0.276 (2)	0.5534 (8)	0.733 (2)	0.088*	
O2	0.22317 (11)	0.60724 (11)	0.53150 (11)	0.0383 (3)	
N1	0.20135 (10)	0.37901 (11)	0.76126 (12)	0.0224 (3)	
N2	0.39950 (10)	0.44243 (10)	0.79467 (11)	0.0203 (3)	
C1	0.22858 (14)	0.66340 (14)	0.62456 (15)	0.0290 (4)	
C2	0.20107 (11)	0.78707 (13)	0.62330 (14)	0.0223 (3)	
C3	0.22051 (12)	0.85085 (14)	0.73285 (15)	0.0252 (3)	
H3	0.2497	0.8147	0.8102	0.030*	
C4	0.19729 (14)	0.96692 (15)	0.72878 (18)	0.0335 (4)	
H4	0.2113	1.0104	0.8033	0.040*	
C5	0.15377 (16)	1.01962 (16)	0.6164 (2)	0.0409 (5)	
H5	0.1376	1.0991	0.6138	0.049*	
C6	0.13392 (15)	0.95635 (17)	0.50764 (19)	0.0388 (4)	
H6	0.1037	0.9926	0.4307	0.047*	
C7	0.15784 (12)	0.84071 (15)	0.51025 (16)	0.0290 (4)	
H7	0.1448	0.7980	0.4352	0.035*	
C8	0.02727 (13)	0.41778 (19)	0.65105 (17)	0.0383 (4)	
H8A	0.0627	0.4698	0.6070	0.058*	
H8B	−0.0154	0.4626	0.6926	0.058*	
H8C	−0.0156	0.3648	0.5916	0.058*	
C9	0.10420 (12)	0.35052 (15)	0.74590 (15)	0.0270 (3)	
C10	0.07329 (13)	0.26372 (16)	0.81689 (16)	0.0320 (4)	
H10	0.0032	0.2450	0.8033	0.038*	
C11	0.14511 (14)	0.20691 (15)	0.90530 (16)	0.0307 (4)	
H11	0.1252	0.1483	0.9535	0.037*	
C12	0.24909 (12)	0.23568 (13)	0.92478 (14)	0.0241 (3)	
C13	0.27308 (11)	0.32317 (12)	0.84953 (13)	0.0202 (3)	
C14	0.32829 (14)	0.18039 (14)	1.01699 (14)	0.0280 (4)	
H14	0.3116	0.1210	1.0668	0.034*	
C15	0.42676 (13)	0.21252 (14)	1.03328 (14)	0.0278 (4)	
H15	0.4784	0.1751	1.0945	0.033*	
C16	0.45439 (12)	0.30185 (13)	0.95988 (14)	0.0225 (3)	
C17	0.37851 (11)	0.35697 (12)	0.86771 (13)	0.0192 (3)	
C18	0.55556 (12)	0.33981 (14)	0.97563 (14)	0.0260 (3)	
H18	0.6092	0.3058	1.0372	0.031*	
C19	0.57615 (12)	0.42575 (14)	0.90205 (15)	0.0261 (3)	
H19	0.6441	0.4520	0.9123	0.031*	
C20	0.49563 (12)	0.47512 (13)	0.81070 (14)	0.0225 (3)	
C21	0.51601 (14)	0.56796 (15)	0.72651 (16)	0.0315 (4)	
H21A	0.4683	0.5603	0.6445	0.047*	
H21B	0.5860	0.5609	0.7186	0.047*	
H21C	0.5071	0.6433	0.7615	0.047*	

### Atomic displacement parameters (Å^2^)

**Table:**

	*U*^11^	*U*^22^	*U*^33^	*U*^12^	*U*^13^	*U*^23^
O1	0.1251 (15)	0.0251 (7)	0.0210 (6)	0.0281 (8)	0.0100 (8)	0.0043 (5)
O2	0.0607 (9)	0.0301 (7)	0.0209 (6)	0.0070 (6)	0.0049 (6)	−0.0025 (5)
N1	0.0235 (6)	0.0242 (6)	0.0198 (6)	0.0004 (5)	0.0060 (5)	−0.0013 (5)
N2	0.0254 (6)	0.0179 (6)	0.0175 (6)	−0.0008 (5)	0.0054 (5)	−0.0013 (4)
C1	0.0404 (9)	0.0242 (8)	0.0206 (7)	0.0036 (7)	0.0046 (7)	0.0022 (6)
C2	0.0206 (7)	0.0234 (7)	0.0234 (7)	0.0014 (5)	0.0065 (6)	0.0042 (6)
C3	0.0262 (7)	0.0261 (8)	0.0251 (8)	0.0012 (6)	0.0096 (6)	0.0023 (6)
C4	0.0398 (9)	0.0256 (8)	0.0416 (10)	0.0008 (7)	0.0221 (8)	−0.0022 (7)
C5	0.0503 (11)	0.0248 (8)	0.0562 (12)	0.0117 (8)	0.0293 (10)	0.0124 (8)
C6	0.0396 (10)	0.0386 (10)	0.0408 (10)	0.0120 (8)	0.0150 (8)	0.0197 (8)
C7	0.0262 (8)	0.0337 (9)	0.0268 (8)	0.0031 (6)	0.0063 (6)	0.0078 (6)
C8	0.0252 (8)	0.0568 (12)	0.0318 (9)	0.0056 (8)	0.0050 (7)	0.0026 (8)
C9	0.0248 (8)	0.0330 (8)	0.0235 (8)	0.0002 (6)	0.0067 (6)	−0.0058 (6)
C10	0.0292 (8)	0.0389 (10)	0.0305 (9)	−0.0096 (7)	0.0124 (7)	−0.0077 (7)
C11	0.0391 (9)	0.0296 (8)	0.0273 (8)	−0.0099 (7)	0.0155 (7)	−0.0029 (6)
C12	0.0341 (8)	0.0205 (7)	0.0193 (7)	−0.0034 (6)	0.0098 (6)	−0.0022 (6)
C13	0.0264 (7)	0.0184 (7)	0.0164 (6)	−0.0008 (5)	0.0066 (6)	−0.0032 (5)
C14	0.0424 (9)	0.0213 (7)	0.0209 (7)	−0.0022 (6)	0.0089 (7)	0.0029 (6)
C15	0.0382 (9)	0.0234 (8)	0.0199 (7)	0.0055 (6)	0.0039 (7)	0.0036 (6)
C16	0.0285 (8)	0.0210 (7)	0.0173 (7)	0.0039 (6)	0.0046 (6)	−0.0019 (5)
C17	0.0254 (7)	0.0172 (6)	0.0152 (6)	0.0009 (5)	0.0055 (6)	−0.0026 (5)
C18	0.0252 (7)	0.0285 (8)	0.0220 (7)	0.0064 (6)	0.0016 (6)	−0.0020 (6)
C19	0.0221 (7)	0.0302 (8)	0.0267 (8)	0.0004 (6)	0.0073 (6)	−0.0054 (6)
C20	0.0274 (7)	0.0213 (7)	0.0201 (7)	−0.0012 (6)	0.0086 (6)	−0.0048 (5)
C21	0.0354 (9)	0.0294 (9)	0.0315 (9)	−0.0072 (7)	0.0121 (7)	0.0008 (7)

### Geometric parameters (Å, °)

**Table:**

O1—C1	1.317 (2)	C9—C10	1.412 (2)
O1—H1O	0.839 (12)	C10—C11	1.367 (3)
O2—C1	1.213 (2)	C10—H10	0.9500
N1—C9	1.328 (2)	C11—C12	1.412 (2)
N1—C13	1.3597 (19)	C11—H11	0.9500
N2—C20	1.327 (2)	C12—C13	1.410 (2)
N2—C17	1.3614 (19)	C12—C14	1.436 (2)
C1—C2	1.487 (2)	C13—C17	1.448 (2)
C2—C7	1.394 (2)	C14—C15	1.355 (2)
C2—C3	1.396 (2)	C14—H14	0.9500
C3—C4	1.386 (2)	C15—C16	1.432 (2)
C3—H3	0.9500	C15—H15	0.9500
C4—C5	1.385 (3)	C16—C17	1.410 (2)
C4—H4	0.9500	C16—C18	1.410 (2)
C5—C6	1.385 (3)	C18—C19	1.367 (2)
C5—H5	0.9500	C18—H18	0.9500
C6—C7	1.384 (3)	C19—C20	1.411 (2)
C6—H6	0.9500	C19—H19	0.9500
C7—H7	0.9500	C20—C21	1.503 (2)
C8—C9	1.502 (2)	C21—H21A	0.9800
C8—H8A	0.9800	C21—H21B	0.9800
C8—H8B	0.9800	C21—H21C	0.9800
C8—H8C	0.9800		
			
C1—O1—H1O	108 (2)	C10—C11—C12	119.76 (15)
C9—N1—C13	118.52 (14)	C10—C11—H11	120.1
C20—N2—C17	118.58 (13)	C12—C11—H11	120.1
O2—C1—O1	124.10 (16)	C13—C12—C11	117.00 (15)
O2—C1—C2	123.66 (15)	C13—C12—C14	120.36 (15)
O1—C1—C2	112.24 (14)	C11—C12—C14	122.64 (15)
C7—C2—C3	119.61 (15)	N1—C13—C12	122.99 (14)
C7—C2—C1	119.28 (15)	N1—C13—C17	118.06 (13)
C3—C2—C1	121.09 (14)	C12—C13—C17	118.94 (14)
C4—C3—C2	120.00 (15)	C15—C14—C12	120.29 (15)
C4—C3—H3	120.0	C15—C14—H14	119.9
C2—C3—H3	120.0	C12—C14—H14	119.9
C5—C4—C3	120.17 (17)	C14—C15—C16	121.19 (15)
C5—C4—H4	119.9	C14—C15—H15	119.4
C3—C4—H4	119.9	C16—C15—H15	119.4
C4—C5—C6	119.92 (17)	C17—C16—C18	117.04 (14)
C4—C5—H5	120.0	C17—C16—C15	119.87 (15)
C6—C5—H5	120.0	C18—C16—C15	123.08 (14)
C7—C6—C5	120.49 (17)	N2—C17—C16	122.89 (14)
C7—C6—H6	119.8	N2—C17—C13	117.76 (13)
C5—C6—H6	119.8	C16—C17—C13	119.34 (13)
C6—C7—C2	119.82 (17)	C19—C18—C16	119.79 (14)
C6—C7—H7	120.1	C19—C18—H18	120.1
C2—C7—H7	120.1	C16—C18—H18	120.1
C9—C8—H8A	109.5	C18—C19—C20	119.44 (15)
C9—C8—H8B	109.5	C18—C19—H19	120.3
H8A—C8—H8B	109.5	C20—C19—H19	120.3
C9—C8—H8C	109.5	N2—C20—C19	122.25 (14)
H8A—C8—H8C	109.5	N2—C20—C21	117.01 (14)
H8B—C8—H8C	109.5	C19—C20—C21	120.74 (14)
N1—C9—C10	122.31 (16)	C20—C21—H21A	109.5
N1—C9—C8	116.68 (15)	C20—C21—H21B	109.5
C10—C9—C8	120.99 (15)	H21A—C21—H21B	109.5
C11—C10—C9	119.42 (15)	C20—C21—H21C	109.5
C11—C10—H10	120.3	H21A—C21—H21C	109.5
C9—C10—H10	120.3	H21B—C21—H21C	109.5
			
O2—C1—C2—C7	6.1 (3)	C14—C12—C13—C17	−0.6 (2)
O1—C1—C2—C7	−174.24 (17)	C13—C12—C14—C15	0.6 (2)
O2—C1—C2—C3	−172.06 (17)	C11—C12—C14—C15	−178.93 (15)
O1—C1—C2—C3	7.6 (2)	C12—C14—C15—C16	0.1 (2)
C7—C2—C3—C4	−0.3 (2)	C14—C15—C16—C17	−0.8 (2)
C1—C2—C3—C4	177.84 (15)	C14—C15—C16—C18	178.49 (15)
C2—C3—C4—C5	0.7 (3)	C20—N2—C17—C16	0.2 (2)
C3—C4—C5—C6	−0.4 (3)	C20—N2—C17—C13	179.49 (12)
C4—C5—C6—C7	−0.4 (3)	C18—C16—C17—N2	0.7 (2)
C5—C6—C7—C2	0.8 (3)	C15—C16—C17—N2	−179.97 (13)
C3—C2—C7—C6	−0.5 (2)	C18—C16—C17—C13	−178.60 (13)
C1—C2—C7—C6	−178.63 (16)	C15—C16—C17—C13	0.7 (2)
C13—N1—C9—C10	−0.7 (2)	N1—C13—C17—N2	−0.3 (2)
C13—N1—C9—C8	177.54 (14)	C12—C13—C17—N2	−179.36 (13)
N1—C9—C10—C11	0.4 (3)	N1—C13—C17—C16	179.01 (13)
C8—C9—C10—C11	−177.79 (16)	C12—C13—C17—C16	0.0 (2)
C9—C10—C11—C12	0.1 (2)	C17—C16—C18—C19	−0.6 (2)
C10—C11—C12—C13	−0.3 (2)	C15—C16—C18—C19	−179.93 (15)
C10—C11—C12—C14	179.27 (15)	C16—C18—C19—C20	−0.3 (2)
C9—N1—C13—C12	0.6 (2)	C17—N2—C20—C19	−1.1 (2)
C9—N1—C13—C17	−178.42 (13)	C17—N2—C20—C21	178.74 (13)
C11—C12—C13—N1	−0.1 (2)	C18—C19—C20—N2	1.2 (2)
C14—C12—C13—N1	−179.61 (14)	C18—C19—C20—C21	−178.67 (14)
C11—C12—C13—C17	178.91 (13)		

### Hydrogen-bond geometry (Å, °)

**Table:**

Cg is the centroid of the C2–C7 ring.

**Table:**

*D*—H···*A*	*D*—H	H···*A*	*D*···*A*	*D*—H···*A*
O1—H1o···N1	0.839 (12)	2.327 (16)	2.973 (2)	134 (2)
O1—H1o···N2	0.839 (12)	2.09 (2)	2.788 (2)	141 (2)
C19—H19···Cg^i^	0.95	2.60	3.426 (2)	145

Symmetry codes:  (i) −*x*+1, *y*−1/2, −*z*+3/2.
